# Supplementary material for: Empirical methods for controlling false positives and estimating confidence in ChIP-Seq peaks
Source: BMC Bioinformatics. 2008 Dec 5;9:523. doi: 10.1186/1471-2105-9-523 (PMC2628906; doi:10.1186/1471-2105-9-523)
Supplement: Additional File 1 — A variety of html documents from the USeq web site detailing the available applications, their best usage, output file type descriptions, command line menus, etc. [file 1471-2105-9-523-S1.zip › USeqUserGuides/JavaDoc.zip!/JavaDoc/edu/utah/seq/data/SmoothingWindowInfo.html]

SmoothingWindowInfo


|  |  |  |
| --- | --- | --- |
| **Overview** \| **Package** \| **Class** \| **Use** \| **Tree** \| **Deprecated** \| **Index** \| **Help** | |  |
| **PREV CLASS**   **NEXT CLASS** | **FRAMES**    **NO FRAMES**     **All Classes** |  |
| SUMMARY: NESTED \| FIELD \| CONSTR \| METHOD | DETAIL: FIELD \| CONSTR \| METHOD |  |


---


## edu.utah.seq.data Class SmoothingWindowInfo

```
java.lang.Object
  edu.utah.seq.data.SmoothingWindowInfo
```

**All Implemented Interfaces:**: java.io.Serializable

---

``` public class SmoothingWindowInfo extends java.lang.Object implements java.io.Serializable ```

Chromosome specific container for window level results from EnrichedRegionMaker.

**See Also:**: Serialized Form

---

| **Field Summary** | |
| --- | --- |
| `static long` | `serialVersionUID` |


**Constructor Summary**

`SmoothingWindowInfo(SmoothingWindow[] sm,
Info info)`


| **Method Summary** | |
| --- | --- |
| `Info` | `getInfo()` |
| `SmoothingWindow[]` | `getSm()` |
| `static float[]` | `minMax(SmoothingWindowInfo[] swi, int scoreIndex)`             Returns min and max of the scores for a given scoreIndex. |
| `void` | `setInfo(Info info)` |
| `void` | `setSm(SmoothingWindow[] sm)` |

**Methods inherited from class java.lang.Object**

`equals, getClass, hashCode, notify, notifyAll, toString, wait, wait, wait`

**Field Detail**

### serialVersionUID

```
public static final long serialVersionUID
```

**See Also:**: Constant Field Values


**Constructor Detail**

### SmoothingWindowInfo

```
public SmoothingWindowInfo(SmoothingWindow[] sm,
                           Info info)
```


**Method Detail**

### minMax

```
public static float[] minMax(SmoothingWindowInfo[] swi,
                             int scoreIndex)
```

:   Returns min and max of the scores for a given scoreIndex.

---


### getInfo

```
public Info getInfo()
```

---


### setInfo

```
public void setInfo(Info info)
```

---


### getSm

```
public SmoothingWindow[] getSm()
```

---


### setSm

```
public void setSm(SmoothingWindow[] sm)
```


---


|  |  |  |
| --- | --- | --- |
| **Overview** \| **Package** \| **Class** \| **Use** \| **Tree** \| **Deprecated** \| **Index** \| **Help** | |  |
| **PREV CLASS**   **NEXT CLASS** | **FRAMES**    **NO FRAMES**     **All Classes** |  |
| SUMMARY: NESTED \| FIELD \| CONSTR \| METHOD | DETAIL: FIELD \| CONSTR \| METHOD |  |


---
